# Evaluation of a SiPM array detector coupled to a LFS-3 pixellated scintillator for PET/MR applications

**DOI:** 10.1186/2197-7364-2-S1-A9

**Published:** 2015-05-18

**Authors:** Stratos David, Eleftherios Fysikopoulos, Maria Georgiou, George Loudos

**Affiliations:** Technological Educational Institute of Athens, Greece; Department of Medical School, University of Thessaly, Larissa, Greece

SiPM arrays are insensitive to magnetic fields and thus good candidates for hybrid PET/MR imaging systems. Moreover, due to their small size and flexibility can be used in dedicated small field of view small animal imaging detectors and especially in head PET/MR studies in mice. Co-doped LFS-3 scintillator crystals have higher light yield and slightly faster response than that of LSO:Ce mainly due to the co-doped activation of emission centers with varying materials such as Ce, Gd, Sc, Y, La, Tb, or Ca distributed at the molecular scale through the lutetium silicate crystal host. The purpose of this study is to investigate the behavior of the SensL ArraySL-4 (4x4 element array of 3x3 mm^2^ silicon photomultipliers) optical detector coupled to a 6x6 LFS-3 scintillator array, with 2x2x5 mm^3^ crystal size elements, for possible applications in small field of view PET/MR imaging detectors. We have designed a symmetric resistive charge division circuit to read out the signal outputs of 4x4 pixel SiPM array reducing the 16 pixel outputs of the photodetector to 4 position signals. The 4 position signals were digitized using free running Analog to Digital Converters. The ADCs sampling rate was 50 MHz. An FPGA (Spartan 6 LX150T) was used for triggering and digital signal processing of the pulses. Experimental evaluation was carried out with ^22^Na radioactive source and the parameters studied where energy resolution and peak to valley ratio. The first preliminary results of the evaluation shows a clear visualization of the discrete 2x2x5 mm^3^ LFS-3 scintillator elements. The mean peak to valley ratio of the horizontal profiles on the raw image was measured equal to 11 while the energy resolution was calculated equal to 30% at the central pixels.

